# Sulfatinib, a novel multi-targeted tyrosine kinase inhibitor of FGFR1, CSF1R, and VEGFR1–3, suppresses osteosarcoma proliferation and invasion *via* dual role in tumor cells and tumor microenvironment

**DOI:** 10.3389/fonc.2023.1158857

**Published:** 2023-06-08

**Authors:** Song Liao, Jianxiong Li, Song Gao, Yuchen Han, Xinli Han, Yanan Wu, Jingyou Bi, Meng Xu, Wenzhi Bi

**Affiliations:** ^1^ Medical School of Chinese PLA, Beijing, China; ^2^ Senior Department of Orthopedics, The Fourth Medical Center, Chinese PLA General Hospital, Beijing, China

**Keywords:** sulfatinib, osteosarcoma, targeted therapy, tumor microenvironment, cancer-associated fibroblast, tumor-associated macrophage

## Abstract

**Introduction:**

Tumor progression is driven by intrinsic malignant behaviors caused by gene mutation or epigenetic modulation, as well as crosstalk with the components in the tumor microenvironment (TME). Considering the current understanding of the tumor microenvironment, targeting the immunomodulatory stromal cells such as cancer-associated fibroblasts (CAFs) and tumor-associated macrophages (TAMs) could provide a potential therapeutic strategy. Here, we investigated the effect of sulfatinib, a multi-targeted tyrosine kinase inhibitor (TKI) of FGFR1, CSF1R, and VEGFR1–3, on the treatment of osteosarcoma (OS).

**Methods:**

In vitro, the antitumor effect was tested by clony formation assay and apoptosis assay.The inhibition of tumor migration and invasion was detected by Transwell assay, and the de-polarization of macrophage was detected by flow cytometry.In vivo, subcutaneous and orthotopic tumor models were established to verify antitumor effect, and the underlying mechanism was verified by immunohistochemistry(IHC), immunofluorescence(IF) and flow cytometry.

**Results:**

Sulfatinib suppressed OS cell migration and invasion by inhibiting epithelial–mesenchymal transition (EMT) by blocking the secretion of basic fibroblast growth factor (bFGF) in an autocrine manner. In addition, it regulated immune TME via inhibition of the migration of skeletal stem cells (SSCs) to the TME and the differentiation from SSCs to CAFs. Moreover, sulfatinib can suppress OS by modulation of the TME by inhibiting M2 polarization of macrophages. Systemic treatment of sulfatinib can reduce immunosuppression cells M2-TAMs, Tregs, and myeloid-derived suppressor cells (MDSCs) and increase cytotoxic T-cell infiltration in tumors, the lungs, and the spleens.

**Discussion:**

Our preclinical experiments have shown that sulfatinib can inhibit the proliferation, migration, and invasion of OS by playing a dual role on tumor cells and the tumor microenvironment simultaneously and systematically reverse immunosuppression to immune activation status, which could be translated into clinical trials.

## 1 Introduction

Osteosarcoma (OS) is the most common primary malignant bone tumor in children and adolescents and has a poor prognosis due to its high malignancy, early metastasis, and rapid progression (1). The current standard treatment for osteosarcoma is neoadjuvant chemotherapy combined with surgery, and the 5-year overall survival rate is approximately 60%–70% (2). For metastatic disease, complete surgical resection of all metastases to achieve tumor-free status is essential to survival. In the setting of unresectable osteosarcoma, chemotherapy, targeted therapy, immunotherapy, or combined treatment may be adopted (3). However, there is no effective second-line treatment for metastatic osteosarcoma. Survival for osteosarcoma patients with metastasis at initial diagnosis has remained unchanged over the past 30 years, with an overall 5-year survival rate of only approximately 20% (3, 4). Therefore, the treatment of refractory osteosarcoma remains a clinical challenge.

According to the origin, differentiation, and biological behaviors of osteosarcoma, several subtypes are divided. Even though conventional osteosarcoma is the most common subtype, its origin and differentiation are not completely consistent (5). The occurrence and development of osteosarcoma are a complex process involving gene mutations, epigenetic modifications, angiogenesis, and remodeling of the tumor microenvironment (6). Currently, anti-tumor angiogenesis inhibitors, such as sorafenib and regorafenib, have been applied and shown good clinical efficacy in osteosarcoma, as angiogenesis is a major event involved in the entire process of tumor development (7, 8). In addition, molecular targeted therapy has shown promising translational applications for locally aggressive bone sarcomas with intermediate malignant behavior such as giant cell tumor of bone (GCTB) and desmoplastic fibroma (DF) (9). However, tumors can be studied as “wounds that fail to heal” with damage and repair occurring simultaneously (10). Cytokine fibroblast growth factor (FGF) and its receptors (FGFRs) are involved in promoting cell proliferation, angiogenesis, migration, and invasion, which has been revealed in numerous tumor settings including liver cancer and pancreatic cancer (11, 12). Similar to findings in osteosarcoma, FGFR1 is over- expressed in the majority of the osteosarcoma tissue samples, and it is associated with a high incidence of metastasis and poor prognosis (13). These findings indicate that FGFR1 amplification is involved in the pathogenesis of osteosarcoma and that FGFR1 inhibitors are one of the promising strategies for osteosarcoma treatment (14). Meanwhile, FGFR1 and other angiogenic receptors are also one of the underlying mechanisms of anti-angiogenic resistance (15). Therefore, the use of multi-targeted tyrosine kinase inhibitors (TKIs) to achieve simultaneous inhibition of these two signaling pathways may enhance the efficacy of osteosarcoma treatment (16).

In recent years, intensive evidence reveals that cancer-associated fibroblasts (CAFs) and tumor-associated macrophages (TAMs) are important components of the tumor microenvironment, which regulates the tumor immune microenvironment by secreting various cytokines and growth factors (17–19). Cancer-associated fibroblasts can alter the extracellular matrix to provide a tumor- supportive microenvironment promoting tumor migration and metastasis (20). Tumor- associated macrophages (or M2 macrophages) also play a tumor-promoting role, which is correlated with low grade and poor prognosis in a variety of malignancies (21, 22). Targeting TAM immunotherapy strategy that repolarizes M2 macrophages to the anti-tumorigenic M1 macrophages is effective for anti-tumor therapy, which is under early- phase investigations in various cancer types (23, 24). Osteosarcoma is a heterogeneous disease adjacent to the bone marrow tissue, which confirmed by single-cell RNA sequencing (scRNA-seq) that CAFs and TAMs are components of the osteosarcoma microenvironment (25, 26). Thus, CAFs and TAMs are both potential target cells in osteosarcoma therapy. A recent study has confirmed that inhibition of TAM polarization plays an important role in suppressing sarcoma, which deserves further research (27).

Blocking the FGF and its receptor can inhibit the production and activation of CAFs (28). Similarly, colony- stimulating factor 1 (CSF1) and its receptor CSF1R play an important role in the recruitment and differentiation of macrophages (29). Sulfatinib is a multi-targeted TKI that targets FGFR1, CSF1R, and vascular endothelial growth factor receptors (VEGFR1–3). The efficacy of sulfatinib has been evaluated in phase I clinical trials on extra pancreatic neuroendocrine tumors (30). To explore the therapeutic effect of sulfatinib in osteosarcoma and its underlying mechanism, preclinical animal experiments were conducted in our study for verification, laying a foundation for subsequent clinical trials.

## 2 Materials and methods

### 2.1 Materials

Murine recombinant IL13 (Catalog # PRP100398) and IL4 (Catalog # PRP100498) were purchased from Abbkine (Wuhan, China). Murine recombinant granulocyte-colony stimulation factor (G-CSF) (Catalog # 414-CS) proteins were purchased from R&D (Minnesota, USA). Recombinant human FGF basic (Catalog # 4114-TC) protein and antibody (Catalog # AF-233-NA) were purchased from R&D (Minnesota, USA). Novel TKIs sulfatinib and doxorubicin were purchased from Selleck (Munich, Germany). Drugs were dissolved in dimethyl sulfoxide (DMSO) and stored at −20°C.

### 2.2 Cell lines and cell culture

Human OS cell lines MG63, U2OS, and HOS and murine cell lines K7M2 and RAW264.7 were purchased from Procell (Wuhan, China). Cells were cultured in Dulbecco’s modified Eagle’s medium (DMEM) containing 10% fetal bovine serum (FBS) and 1% penicillin and streptomycin at 37°C in a humidified atmosphere containing 5% CO_2_. All cell lines were confirmed to have no cross- contamination during this study. Cell lines were tested for mycoplasma contamination at least every month, and no contamination was detected.

### 2.3 Preparation of BMDM

Bone marrow cells were isolated and cultured as previously described (31). For briefly, 4- to 6-week-old C57BL/6 mice were sacrificed by cervical dislocation and disinfected in 75% ethanol for 5 min. Bone marrow was harvested from the femur and tibia of mice by flushing with sterile phosphate-buffered saline (PBS). Bone marrow cells differentiated into bone marrow-derived macrophages (BMDMs) with M-CSF (20 ng/ml). After changing the fresh medium on the third and fifth days, BMDMs were induced.

### 2.4 Conditioned medium preparation

MG63, U2OS, and K7M2 cells were seeded at 1 × 10^7^ cells per 15-cm cell culture dish in 20 ml of DMEM containing 10% FBS. The medium was changed 24 h later, and supernatants were collected at 48 h and passed through a 0.22-μm vacuum filter for further experiments.

### 2.5 Skeletal stem cell isolation and cultivation

Human SSCs were isolated from the tumor segment after wide resection of patients with osteosarcoma. The normal bone and tumor fragments were rinsed with 0.1 mol/L PBS and digested for 2 h at 37°C using 0.1% Collagenase II (GIBCO, Grand Island, NY, USA). Then, the bone chips were washed with alpha-minimal essential medium (α-MEM) containing 10% FBS, centrifuged at 500 g for 10 min at 4°C, and washed twice with PBS, after which the fragments were placed in a 5-cm cell culture dish in 8 ml of α-MEM with 10% FBS and 1% penicillin and streptomycin at 37°C in a humidified atmosphere containing 5% CO_2_. The medium was changed every 3 days. After approximately 5–7 days, cells that migrated from the bone chips could be observed. At approximately 80%–90% confluence, the cells were passaged to a 10-cm cell culture dish. For the subsequent experiments, SSCs underwent three to five passages.

For cell identification, flow cytometry and trilineage induction were performed according to previous protocols (32). Briefly, cells were then collected and stained with PE-CD29, PE-CD44, PE-CD73, PE-CD166, FITC-CD45, FITC-CD90, APC-CD31, and APC-CD105 (eBioScience, San Diego, CA, USA) according to the manufacturer’s instructions. To verify the multipotency of SSCs, osteogenic differentiation and adipogenic differentiation were performed. SSCs in passage 3 were cultured in 48-well plates at a density of 3 × 10^3^ cells/well with osteogenic induction medium (Cyagen, Santa Clara, CA, USA; Catalog # HUXMA-90021) and adipogenic induction medium (Cyagen; Catalog # HUXMA-90031), which were replaced every 3 days for approximately 14–21 days. Cells were stained with Alizarin Red at 2 weeks for osteogenic verification and stained with Oil Red O for adipogenic verification.

### 2.6 Cell viability assay

Human OS cell lines MG63, U2OS, and HOS were cultured in 96-well plates at a density of 5,000 cells per well over night and then treated with sulfatinib and/or doxorubicin (DOX) at the indicated concentrations. After incubation for 24 h, the viability of cells after sulfatinib treatment was assessed by CCK8 assay (Dojindo, Kumamoto, Japan), according to the manufacturer’s instructions. For the dose–response curve, cells were incubated for 24 or 48 h according to the arithmetic concentration gradient. Then, the IC50 values (50% inhibition concentration) were calculated using Prism 9.0 (GraphPad, San Diego, CA, USA).

### 2.7 Clone formation assay

Cells were seeded at a density of 500 cells/well in six-well plates and cultured for 24 h. The cells were treated for approximately 14 days (until visible colonies were formed) with 2 μM of sulfatinib or DMSO. The colonies were stained with crystal violet and counted manually using a light microscope.

### 2.8 Apoptosis assay

Cells were cultured at a density of 3 × 10^5^ cells/well in six-well plates for 24 h with sulfatinib at indicated concentrations (2 or 4 μM). Apoptotic cell death was detected by flow cytometry after Annexin V and propidium iodide (PI) staining.

### 2.9 Western blotting

Western blotting analyses were performed as previously described (33). Briefly, cells were lysed in radioimmunoprecipitation assay (RIPA) lysis buffer (CWBIO, Beijing, China; Catalog #CW2333) containing protease and phosphatase inhibitors (Roche, Basel, Switzerland; Catalog #4693159001). Equal amounts of denatured protein extracts were loaded onto a sodium dodecyl sulfate–polyacrylamide gel electrophoresis (SDS-PAGE) gel, transferred to a nitrocellulose filter membrane (Millipore, Billerica, MA, USA; Catalog #HATF00010), and analyzed by immunoblotting. The membranes were incubated with primary antibodies over night at 4°C. The following day, after incubation with horseradish peroxidase (HRP)-conjugated anti-rabbit or anti-mouse secondary antibodies, the proteins were detected by enhanced chemiluminescence (ECL) detection reagents (Thermo Fisher Scientific, Waltham, MA, USA; Catalog #34580).

### 2.10 RT-PCR

Total RNA was extracted from cellular lysates using the RNApure Tissue&Cell Kit (CWBIO; Catalog #CW0560), and 500 ng of total RNA was reverse transcribed into cDNA using the MonScript™ RTIII All-in-One Mix with dsDNase (Monad, Wuhan, China; Catalog #MR05101). Quantitative RT-PCR was performed using KAPA SYBR^®^ FAST qPCR Kits (Kapa Biosystems, Woburn, MA, USA; Catalog # KK4602) on an ABI QuantStudio 5 Real-Time PCR System (Applied Biosystems, Foster City, CA, USA). Primer sequences are provided as follows: *ACTA2*: **F**-AAAAGACAGCTACGTGGGTGA, **R**-GCCATGTTCTATCGGGTACTTC; *FN1*: **F**-CGGTGGCTGTCAGTCAAAG, **R**-AAACCTCGGCTTCCTCCATAA; C*OL1A1*: **F**-GAGGGCCAAGACGAAGACATC, **R**-CAGATCACGTCATCGCACAAC; *GAPDH*: **F**-GGAGCGAGATCCCTCCAAAAT, **R**-GGCTGTTGTCATACTTCTCATGG; *Mrc1*: **F**-CTCTGTTCAGCTATTGGACGC, **R**-CGGAATTTCTGGGATTCAGCTTC; *Gapdh*: **F**-AGGTCGGTGTGAACGGATTTG, **R**-TGTAGACCATGTAGTTGAGGTCA (synthesized by Tsingke Biotechnology, Beijing, China). Expression measurements were calculated using the ΔΔCT method.

### 2.11 ELISA

Cells were cultured at a density of 2 × 10^5^ cells/well in 24-well plates. After 8-h incubation in a 5% CO_2_ incubator, the fresh medium was changed. In the starvation group, no FBS medium was added. In the hypoxia group, cells were incubated in 1% O_2_ conditions. Twenty-four hours later, the supernatant was collected (>200 μl) for cytokine measurements. Human basic fibroblast growth factor (bFGF) and G-CSF proteins were detected using Human bFGF Quantikine ELISA Kit (Catalog # DFB50) and Human G-CSF Quantikine ELISA Kit (Catalog # DCS50) according to the manufacturer’s protocol.

### 2.12 Flow cytometry assay

RAW 264.7 and BMDM cells were treated by grouping. Cells were then collected and stained with PE-F4/80, FITC-CD80, and APC-CD206 antibodies (BioLegend, San Diego, CA, USA) according to the manufacturer’s instructions. After 30-min incubation in the dark, cells were harvested and washed with PBS. The proportion of M1 (CD80+CD206−) and M2 (CD80+CD206+) macrophages were detected by flow cytometry. At least 1 × 10^4^ cells should be analyzed for each sample.

For *ex vivo* flow cytometry of mouse tissues, single-cell suspensions of tumors, the spleens, and the lungs were prepared by grinding tissue through a 70-μm cell strainer followed by red blood cell lysis. Cells were labeled with the following antibodies (BioLegend) diluted as 1:100 in 1% FBS/PBS for 30 min on ice: F4/80-FITC, CD206-PE, CD45-PerCP, CD80-APC, CD86-PE-Cy7, CD11c-BV421, CD4-FITC, FoxP3-PE, CD25-APC, CD8-PE-Cy7, CD3-BV421, CD11b-PE, CD44-APC, CD62L-APC-Cy7, and Gr1-BV510. Then, they were detected according to the manufacturer’s instructions.

### 2.13 Transwell migration and invasion assays

Transwell migration assays were performed using Transwell^®^ chamber inserts (Costar, 8-μm pore size). OS cells (2 × 10^3^ cells) were suspended in 200 μl of serum-free medium and seeded in the upper chamber, and 800 μl of medium with 20% FBS was added to the lower chamber. After 12-h incubation, cells on the upper surface were gently wiped with a cotton bud, and migrated cells adhering to the lower surface were fixed with 4% paraformaldehyde for 15 min and stained with 0.1% crystal violet for 15 min. After being washed and dried, membranes were mounted on a glass slide, and the number of migrated cells was counted using a Nikon light microscope with a 20× objective. *In vitro* invasion assays were performed using the same chamber but coated with Matrigel. After 24-h incubation, fixation and staining of invaded cells were achieved as previously described. Cell motility was determined by counting migrated cells, and cell invasiveness was determined by counting cells that invaded the Matrigel.

### 2.14 Wound healing assay

For the wound healing assay, OS cells were cultured in six-well plates and grown until they achieve 100% confluence. The confluent monolayer was scratched with a sterile 100-μl pipette tip to create a “wound”. Fresh medium containing 4 μM of sulfatinib and/or 100 ng/ml of bFGF was subsequently added to the wells. Wound closure was photographed by phase-contrast microscopy at 0 and 24 h. The percentage of wound closure was evaluated by ImageJ software (National Institutes of Health).

### 2.15 Endothelial cell tube formation assay

Matrigel was added to a 48-well plate (100 μl/well) and incubated at 37°C for 30 min to solidify the Matrigel. Human umbilical vein endothelial cells (HUVECs) (2 × 10^4^ cells/well) were seeded in the Matrigel-pre-coated wells. A culture medium with 50 ng/ml of VEGF and/or 2 μM of sulfatinib was added to each well. The plates were incubated at 37°C for 12–18 h, after which the endothelial tube formation was stained with Calcein AM and photographed using a fluorescence microscope. The total network length was analyzed with the ImageJ Angiogenesis Analyzer plugin.

### 2.16 Animal models

The animal experiments were approved by the Animal Care and Use Committee of the Chinese PLA General Hospital and were performed in accordance with the approved protocol. Male BALB/c nude mice aged 4–6 weeks (Charles River) were housed in a pathogen-free facility in a 12-h light–dark cycle and allowed free access to water and food (temperature, 23°C; humidity, 55%). The cell suspension (5 × 10^6^ cells/100 μl) was subcutaneously injected into the flanks of nude mice, and tumor sizes were monitored every other day. After 1 week, treatments began when the tumor volume reached approximately 100 mm^3^. The mice were randomly separated into four groups (n = 8) and received intraperitoneal injections of DMSO (control) and/or DOX (4 mg kg^−1^ week^−1^). Sulfatinib (2 mg kg^−1^ day^−1^) was administered orally. Tumor volumes were calculated every 4 days using the following standard formula: volume = 0.5 * length * width^2^. After 20 days, mice were sacrificed, and tumors were harvested and recorded.

For the orthotopic osteosarcoma model, K7M2-wt cells stably expressing luciferase (K7M2-Luc) were injected into the right tibia of each mouse (2 × 10^5^ cells/20 μl). One week after injection, the mice were randomly separated into three groups (n = 8). The mice were orally administered 2 or 4 mg/kg of sulfatinib or DMSO daily. Tumor growth and lung metastasis were assessed by bioluminescence imaging (LB983 NC100 system; Berthold Technologies, Bad Wildbad, Germany). Mice were sacrificed after 28 days of treatment, and the posterior limbs with tumors and the lungs were excised for further experiments. Single- cell suspensions of *ex vivo* tumors, the spleens, and the lungs (n = 3) were prepared and analyzed by flow cytometry assay.

### 2.17 Immunohistochemistry and immunofluorescence assay

Tissue samples were fixed in formalin, embedded in paraffin, and cut into 4-μm sections. The primary organs including the heart, liver, spleen, lung, and kidney were stained with hematoxylin and eosin (H&E). Subcutaneous tumor specimens were immunoassayed with PCNA (1:2,000), CD31 (1:100), E-cadherin (1:100), N-cadherin (1:100), FGFR1 (1:100), p-FGFR1 (1:100), and p-VEGFR2 (1:100). Tumor specimens from the tibia were immunoassayed, with F4/80-green, CD206-red, and DAPI staining indicating nuclei. Images were captured using a microscope (Leica, Wetzlar, Germany).

### 2.18 Statistical analysis

Statistical analysis was performed using Prism 9.0 (GraphPad, San Diego, CA, USA). The quantitative data are shown as the mean ± SD. The significance of the differences between groups was assessed by unpaired Student’s t-test or one-way ANOVA. Statistical significance is indicated by *p < 0.05 and ***p < 0.01.

### 2.19 Ethics statement

The human tumor samples used in this study were approved by the institutional ethical review board of the Chinese People’s Liberation Army General Hospital (rapid review and approval of scientific research projects for use of discarded biological materials), and informed consent was obtained from all donors. The investigation was approved by the authors’ institutional review board and conducted in accordance with ethical standards, according to the Declaration of Helsinki and national and international guidelines.

## 3 Results

### 3.1 Sulfatinib inhibits osteosarcoma cell proliferation and induces apoptosis and enhances chemosensitivity to doxorubicin *in vitro* and *in vivo*

**Figure 1:**
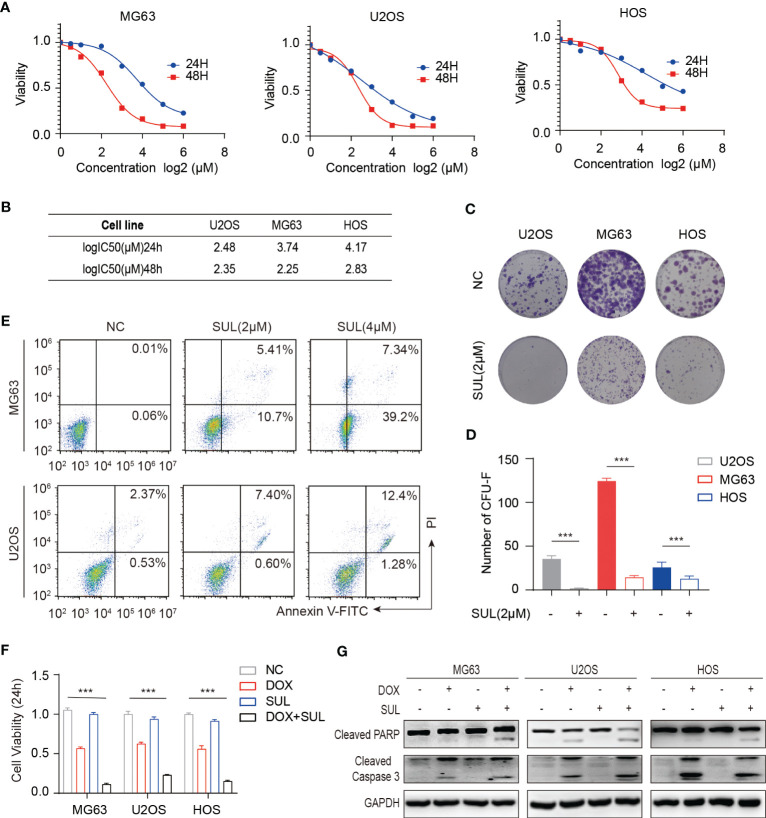
*In vitro*, sulfatinib inhibits osteosarcoma (OS) cell proliferation, induces apoptosis, and enhances chemosensitivity to doxorubicin. **(A, B)** Cell proliferation was measured by CCK8 after incubation with sulfatinib at specified concentrations for 24 and 48 h. Cell viability curves and IC50 values were fitted and calculated. **(C, D)** Colony formation experiments demonstrated that a proper concentration of sulfatinib (2 μM) inhibited OS cell proliferation. The differences were statistically significant (***p < 0.01). **(E)** Annexin V/PI flow cytometry analysis demonstrated that the apoptosis rate of OS cell lines (MG63 and U2OS) increased with the increasing concentration of sulfatinib. **(F)** Cell viability modestly decreased in monotherapy of low- dose (1 μM) sulfatinib but significantly decreased in combination with DOX. **(G)** Western blotting assay confirmed the apoptosis-related protein increased in the combination drug group. DOX, doxorubicin.

Targeted therapy has shown anti-tumor activity in various types of cancer, especially in those with targeting molecules and receptors over-expressed (34, 35). To evaluate the expression of targeted receptors in OS, we performed a pan-cancer analysis of The Cancer Genome Atlas (TCGA) database and found that FGFR1 and CSF1R were highly expressed in sarcomas (**Figures S1A, B**). Meanwhile, sulfatinib has shown anti-tumor activity in various types of cancer including extra pancreatic neuroendocrine tumors and colorectal cancer (30, 36), but its effect on osteosarcoma has not been studied yet. To evaluate the effect of sulfatinib on osteosarcoma, we performed a CCK8 assay at the indicated concentrations for 24 or 48 h in osteosarcoma cell lines HOS, U2OS, and MG63. As a result, the growth curves consistently demonstrated inhibition of sulfatinib on osteosarcoma cell lines in a time- and dose-dependent manner (**Figure 1A**). There was no significant difference in the IC50 values at 48 h of different cell lines; therefore, we used 2 μM of sulfatinib in the subsequent experiments (**Figure 1B**). Here, we performed colony formation experiments on osteosarcoma cell lines with or without the addition of sulfatinib. As the results showed, the colony number was significantly reduced in the sulfatinib group compared with the control group (**Figures 1C, D**). Moreover, to further determine the effect of sulfatinib on apoptosis of osteosarcoma cell lines, we performed Annexin V-PI double staining for quantitative analysis. We found that increasing the concentration of sulfatinib increases the proportions of early and late apoptotic cells in MG63 and U2OS cell lines (**Figure 1E**).

**Figure 2:**
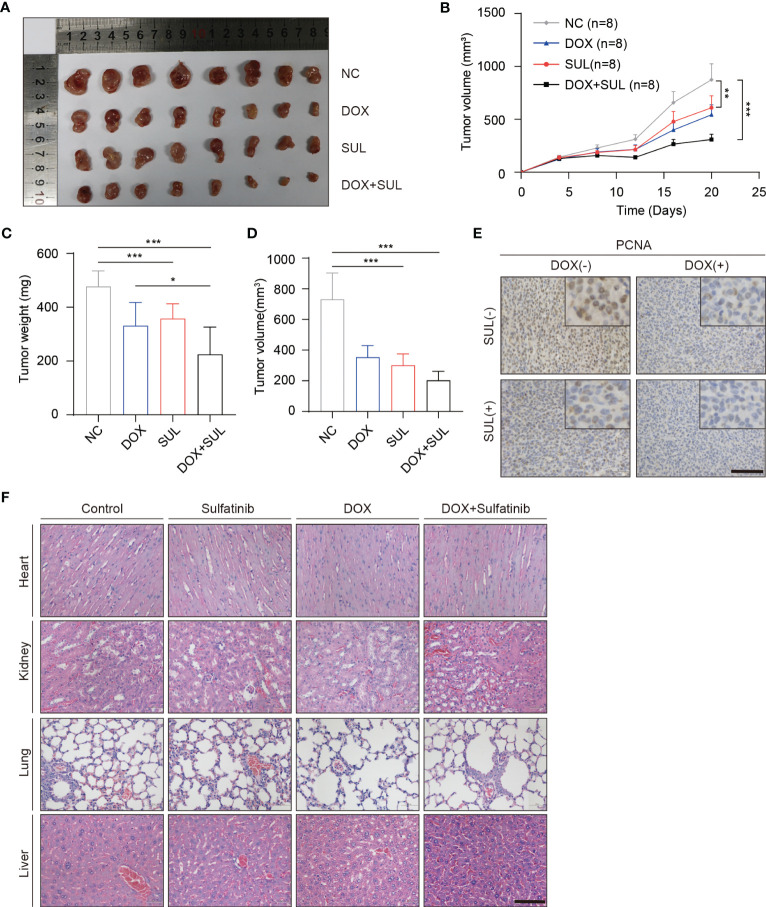
Sulfatinib increases sensitivity to doxorubicin chemotherapy in a subcutaneous xenografted nude mice model. **(A)** Macroscopic appearance of dissected subcutaneous tumors after being treated with different drugs for 20 days. **(B)** Tumor volume of xenograft model of each treatment group (n = 8). **(C, D)** Tumor weight and volume were measured at the end of treatment. Data were demonstrated as mean volume ± SD; comparison between groups was performed by one-way analysis of variance (ANOVA), ***p < 0.01. **(E)** Immunohistochemistry for PCNA in tumor sections after treatment (scale bar, 100 μm). **(F)** Hematoxylin and eosin (H&E) staining showed no abnormalities in major organs after different treatments (scale bar, 100 μm).

Chemotherapeutic resistance frequently develops in osteosarcoma (37); thus, we examined if sulfatinib can enhance the sensitivity to doxorubicin, which is believed to be the most effective chemotherapy agent for osteosarcoma. Therefore, we treated MG63 and U2OS cell lines with doxorubicin alone, sulfatinib alone, or a combination of both drugs for 24 h. Compared with control cells, a low concentration of sulfatinib (1 μM) alone did not obviously affect cell viability; however, it significantly enhanced the inhibitory effect of doxorubicin, reducing the cell viability from 50%–60% to 10%–20% (**Figure 1F**). In addition, we also detected the expression level of apoptosis-related protein and found that the expression of PARP and cleaved caspase 3 was increased in combination with sulfatinib compared with doxorubicin alone (**Figure 1G**). To further verify the effect of sulfatinib *in vivo*, we constructed subcutaneous tumor models in nude mice using U2OS cells. After injection, we randomly divided the mice into four groups: control group with DMSO, DOX treatment group (5 mg/kg, ip, once a week), sulfatinib treatment group (2 mg/kg, orally, daily), and combined treatment group. We recorded the tumor sizes and calculated relative tumor volumes every 4 days to obtain the tumor volume curves (**Figure 2B**). We sacrificed all mice on day 20, completely stripped the tumor tissues, measured the volume and weight of the tumors, and photographed the gross appearance of the tumor. Notably, combined treatment showed a most effective inhibition of tumor growth than any other single- drug treatments (**Figures 2A, C, D**). Moreover, the expression of PCNA in tumor tissues was detected by immunohistochemistry (IHC), and that in the combined treatment group was also profoundly decreased, which further indicated the strongest anti-tumor effect (**Figure 2E**). It reveals that sulfatinib remarkably enhanced chemo-sensitivity to doxorubicin in osteosarcoma treatment. Meanwhile, H&E- stained sections of major organs showed no significant damage, indicating good safety of combined drug administration (**Figure 2F**).

### 3.2 Basic fibroblast growth factor secreted by OS cells in an autocrine manner promotes tumor cell migration and invasion

**Figure 3:**
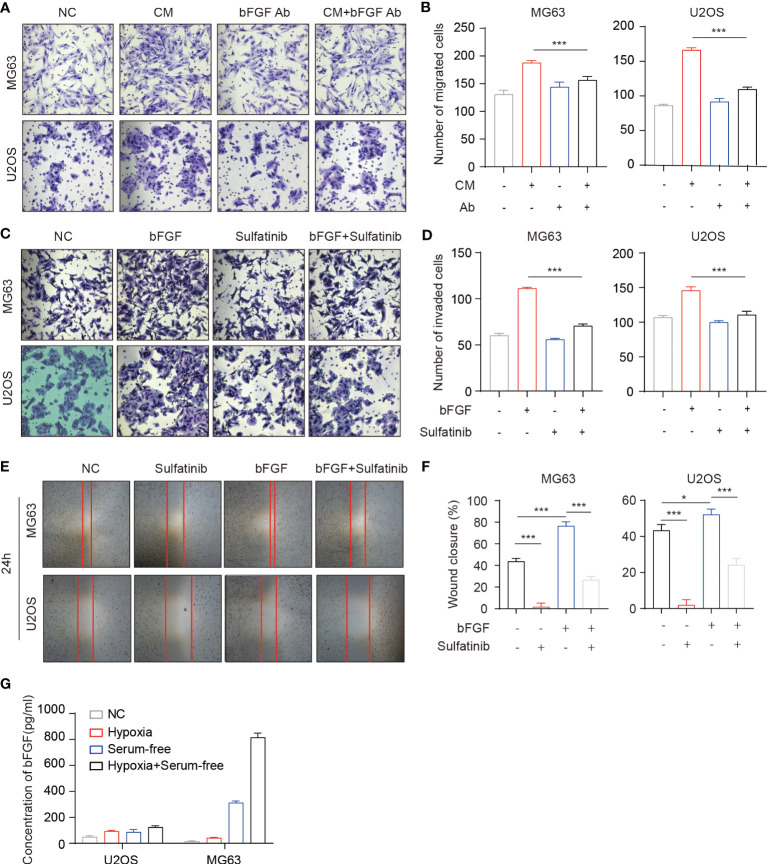
Basic fibroblast growth factor (bFGF) secreted by OS cells in an autocrine manner promotes tumor cell migration and invasion. **(A, B)** Transwell migration assays of OS cells performed with each tumor conditioned medium (CM). Neutralizing antibodies of bFGF significantly decreased migration. **(B)** Number of migrated cells(n = 3, *p < 0.05, ***p<0.01). **(C, D)** Transwell invasion assays of OS cells performed with extrinsic bFGF and sulfatinib. Sulfatinib significantly suppressed migration. **(D)** Number of invaded cells(n = 3, *p < 0.05, ***p<0.01). **(E, F)** Wound healing assays demonstrated that sulfatinib suppressed bFGF- induced cell migration. Relative wound closure rate was quantified (n = 3, *p < 0.05, ***p<0.01). **(G)** The levels of bFGF protein in cell culture supernatants after culture in different conditions for 24 h were assayed by enzyme-linked immunosorbent assay (ELISA). OS, osteosarcoma.

Distant metastasis is the most common failure in osteosarcoma treatment, which involves the interaction of various cells, chemokines, cytokines, and other factors (38). bFGF is a cytokine that can contribute to tumor growth and promote cancer metastasis by affecting the tumor microenvironment (39). To investigate the possible role of bFGF and its effect manner in the migration of OS cells, cell migration was conducted using the conditioned medium from the OS cells (OS-CM) and bFGF neutralized by the anti-bFGF antibody. As expected, bFGF neutralizing antibodies significantly blocked the migration of OS cells induced by OS-CM (**Figures 3A, B**). Therefore, we hypothesized that osteosarcoma cells can secrete bFGF in an autocrine manner. To further verify whether bFGF could be secreted from OS cells, the cells were cultured under normal conditions, in serum-free medium, hypoxic conditions (1% O_2_), and serum-free medium plus hypoxia. After being treated with different conditions as described above, the concentration of bFGF in the culture supernatant was determined by enzyme-linked immunosorbent assay (ELISA). Indeed, we found that bFGF secretion was significantly increased under hypoxia and starvation stimulations, especially in the MG63 cell line (**Figure 3G**). Sulfatinib targets FGFR1, which is highly expressed in osteosarcoma and is a bFGF- binding receptor. For an investigation into the effects of sulfatinib, wound healing assay and Transwell assay were carried out, and the results demonstrated that the migration ability and invasiveness of OS cells enhanced by bFGF were significantly inhibited by sulfatinib (**Figures 3C–F**).

### 3.3 Sulfatinib suppresses OS cell migration and invasion through inhibition of epithelial–mesenchymal transition

**Figure 4:**
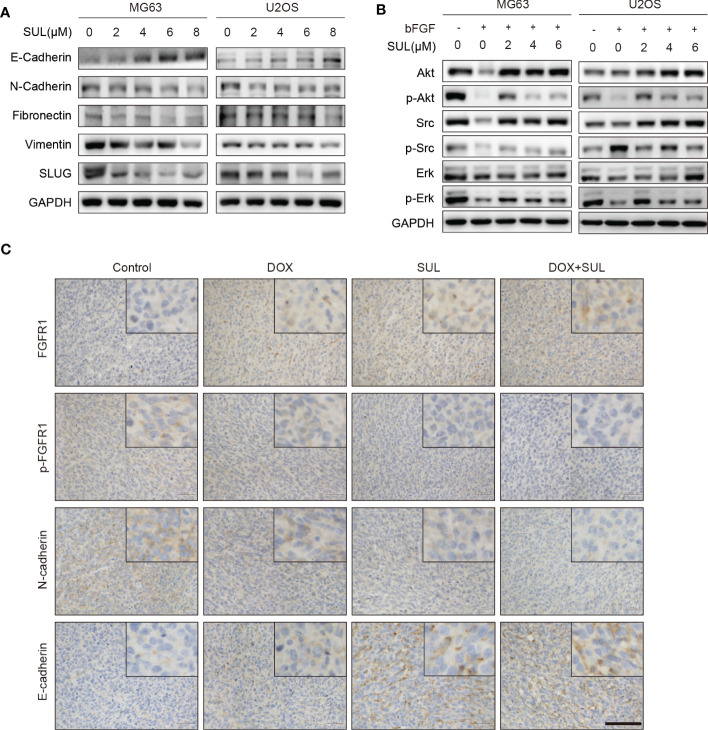
Sulfatinib inhibits OS migration through suppression of epithelial–mesenchymal transition (EMT). **(A)** The protein levels of EMT and metastatic markers were detected by Western blotting after 24 h sulfatinib treatment. **(B)** Sulfatinib inhibits phosphorylation of migration-related signal pathway induced by bFGF. **(C)** Immunohistochemistry for EMT- related markers (FGFR1, p-FGFR1, N-cadherin, and E-cadherin) in tumor sections after treatment (scale bar, 100 μm). OS, osteosarcoma; bFGF, basic fibroblast growth factor.

To explore the underlying mechanism by which FGFR1 inhibition suppresses migration and invasion of OS cells, MG63 and U2OS cells were treated with different concentrations of sulfatinib (2, 4, 6, and 8 μM) for 24 h, and the expression of epithelial–mesenchymal transition (EMT)-related proteins was detected. Western blotting results demonstrated that the expression of the epithelial marker E-cadherin increased, while the expression of mesenchymal markers N-cadherin, Fibronectin, Vimentin, and Slug gradually decreased in a dose-dependent manner with increasing sulfatinib concentrations (**Figure 4A**). We next investigate the effect of sulfatinib treatment on bFGF- induced signaling pathway. Similarly, in response to EMT molecular marker changes, bFGF-induced AKT, SRC, and ERK phosphorylation were gradually decreased with increasing sulfatinib dose, suggesting that sulfatinib suppresses downstream kinases in bFGF- induced EMT pathway (**Figure 4B**). Moreover, in xenograft tumors, sulfatinib monotreatment and chemotherapy combination treatment also effectively suppressed EMT via inhibition of FGFR1 phosphorylation as observed by decreased expression in p-FGFR1 and N-cadherin and increased expression in FGFR1 and E-cadherin determined by the *ex vivo* tissue IHC assay (**Figure 4C**). To sum up, sulfatinib inhibited the bFGF- induced EMT process, thereby suppressing migration and invasion *in vitro* and *in vivo*.

### 3.4 Sulfatinib suppress skeletal stem cells migrated to tumor microenvironment and differentiated into cancer-associated fibroblasts

**Figure 5:**
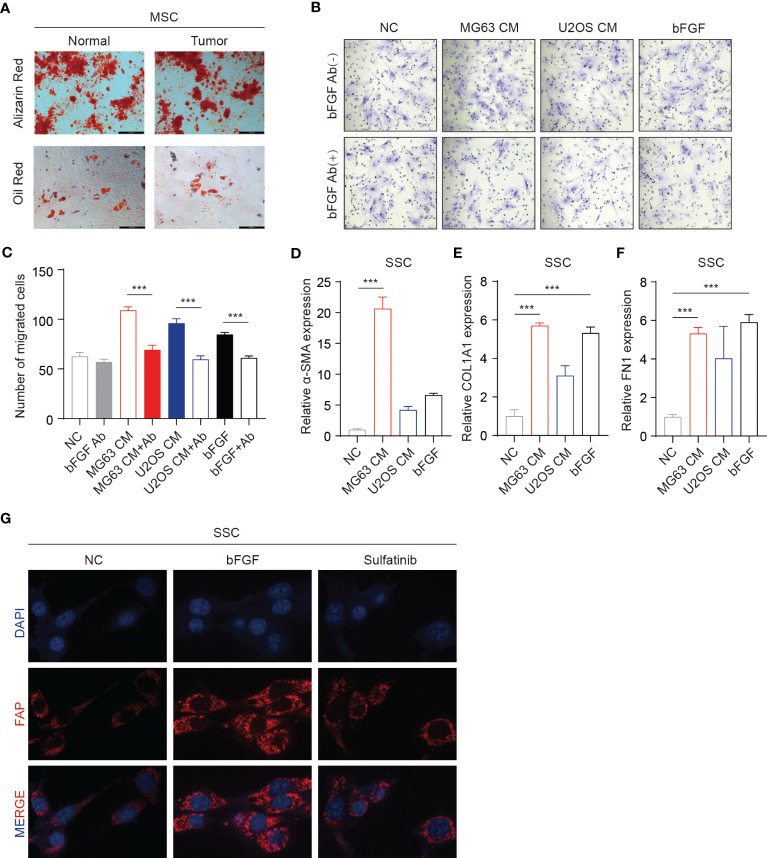
Sulfatinib suppresses skeletal stem cell (SSC) migration and differentiation into cancer-associated fibroblasts (CAFs). **(A)** SSCs isolated from the tumor tissue and adjacent normal bone have the same differentiation potential (scale bar, 100 μm). **(B, C)** Transwell migration assays of SSCs performed with tumor conditioned media (CM). Neutralizing antibodies of bFGF significantly decreased migration. **(C)** Number of migrated cells(n = 3, ***p<0.01). **(D–F)** OS-CM and bFGF increased mRNA expression of CAF-related genes *ACTA2*, *COL1A1*, and *FN1* in SSCs (***p<0.01). **(G)** IF assay showed that sulfatinib inhibited the expression of FAP. OS, osteosarcoma; bFGF, basic fibroblast growth factor; IF, immunofluorescence.

Bone marrow is a vital component of the bone microenvironment and tumor microenvironment, as osteosarcoma derives from and invades the bone. The development of a tumor resembles the constant state of “chronic inflammation”, likened to wounds that never heal. Under abnormal conditions, such as wound healing and tumorigenesis, bFGF and vascular endothelial growth factor (VEGF) promote cytokines, which facilitate the proliferation, differentiation, and migration of endothelial and stroma cells. Thus, bFGF not only plays a role in OS cells but also can recruit mesenchymal stem cells and promote differentiation into CAFs, thus supporting tumor growth and invasiveness and latent further metastasis. To investigate the source of CAFs in the OS–tumor microenvironment (TME), we isolated skeletal stem cells (SSCs) from the tumor tissue and adjacent normal bone, which verified that the same markers and differentiation potential may be speculated to be the same stem cells (**Figure 5A**, **Figures S2A, B**). Furthermore, in the Transwell experiment, SSCs migration was significantly increased in the conditioned medium from OS cells while reduced by the addition of bFGF neutralizing antibodies (**Figures 5B, C**). Moreover, to confirm the transition of SSCs to CAFs, as evaluated by RT-PCR analyses of mRNA expression of CAF-related genes, *ACTA2*, *COL1A1*, and *FN1*, the expression was significantly increased upon exogenous bFGF culture condition and decreased upon addition of sulfatinib (**Figures 5D–F**). Similarly, immunofluorescence for human fibroblast protein (FAP), which is a marker of active fibroblasts, phenotypically revealed that sulfatinib could inhibit the differentiation of SSCs into CAFs and its activation (**Figure 5G**). Taken together, bFGF from OS-TME is a vital factor that modulates cell migration and differentiation, one of which is the induction of SSCs differentiated into CAFs, supporting tumor growth and invasiveness. Sulfatinib could suppress this process, suggesting its therapeutic potential to target CAFs in the OS microenvironment.

### 3.5 Sulfatinib suppresses vascular endothelial growth factor- induced angiogenesis

**Figure 6:**
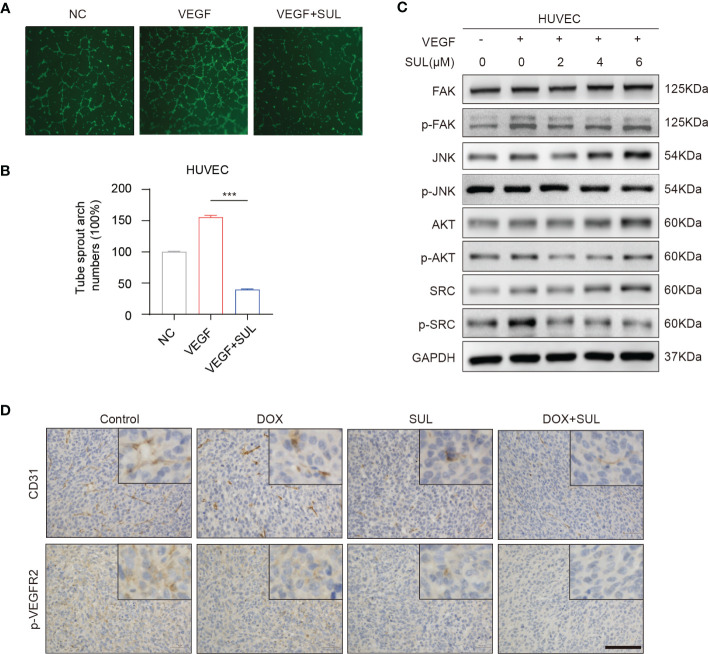
Sulfatinib suppresses vascular endothelial growth factor (VEGF)- induced angiogenesis. **(A)** Sulfatinib inhibits VEGF-induced endothelial tube formation. Representative images of tube formation were demonstrated. Scale bar, 100 μm. **(B)** Relative tube sprout arch numbers are depicted in the histogram (n = 3, ***p < 0.01). **(C)** Sulfatinib inhibits phosphorylation of angiogenesis- related signal pathway induced by VEGF. **(D)** Immunohistochemistry for angiogenesis marker (p-VEGFR2 and CD31) in tumor sections after treatment. Scale bar, 100 μm.

Sulfatinib is a multi- targeted TKI that also inhibits vascular endothelial growth factor receptors 1–3 (VEGFR1–3), whereas the above receptors are not expressed in osteosarcoma. Therefore, sulfatinib cannot directly inhibit OS cell proliferation and invasion. VEGF is known to be a pro-angiogenic factor that stimulates angiogenesis and plays an important role in tumor proliferation and migration. We performed a two-dimensional tube- forming experiment to evaluate the inhibition of sulfatinib on angiogenesis. As a result, sulfatinib can significantly suppress angiogenesis through diminishing VEGF- induced tube formation of HUVECs on Matrigel (**Figures 6A, B**). Furthermore, to verify the underlying mechanism, HUVECs were cultured with various concentrations of sulfatinib in the absence or presence of VEGF for 24 h, and then angiogenesis-related protein expression was detected by Western blotting assay. It was shown that sulfatinib could not only suppress VEGF- induced tube formation but also inhibit the phosphorylation of angiogenesis-related proteins such as FAK, JNK, AKT, and SRC in HUVECs, with a dose-dependent suppressing effect (**Figure 6C**). In addition, *ex vivo* histopathology evaluation (IHC) of xenograft tumors confirmed that the expression of p-VEGFR2 and CD31 was significantly decreased in the sulfatinib monotherapy and combined treatment groups (**Figure 6D**). Ultimately, sulfatinib suppresses tumor angiogenesis by inhibiting the phosphorylation of VEGFR2 and its downstream signaling kinases.

### 3.6 Sulfatinib suppress macrophage polarization via CSF1/CSF1R signaling pathway *in vitro*

**Figure 7:**
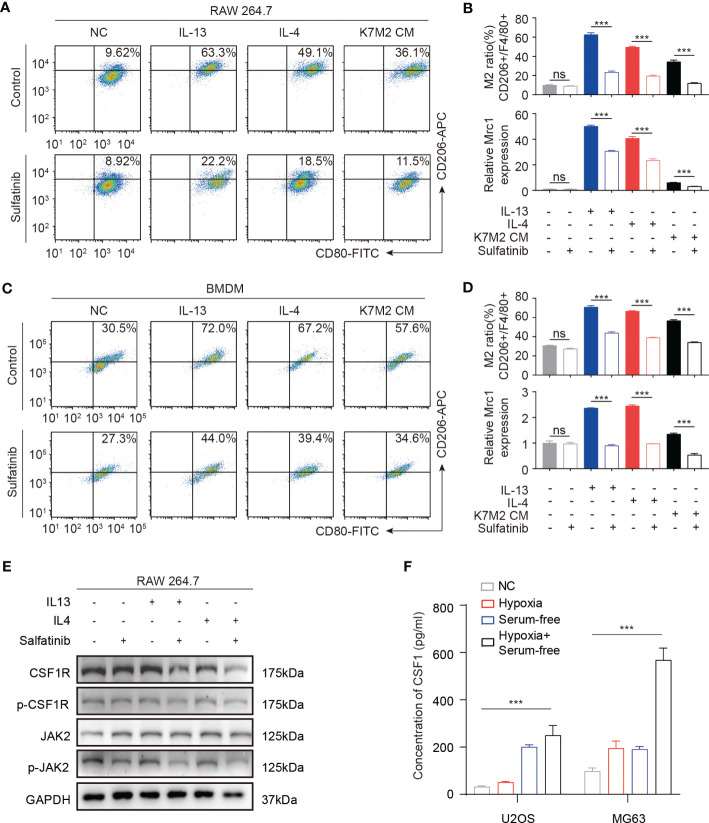
Sulfatinib suppresses macrophage polarization *in vitro*. **(A)** In RAW264.7 macrophage cell line, sulfatinib suppressed interleukin (IL13 and IL4) and K7M2-CM- induced M1-to- M2 macrophage polarization. **(B)** M2 (CD206+) macrophage cell ratio was depicted in the histogram. The mRNA levels of *Mrc1* in RAW264.7 cells treated with IL13, IL4, and K7M2 CM alone or with 2 μM of sulfatinib. Data represent mean ± SD from three independent experiments. ns, not significant; ***p < 0.01 by an unpaired two-tailed *t*- test (*vs.* untreated). **(C)** In BMDM cells, sulfatinib suppressed IL13, IL4, and K7M2-CM- induced M1-to- M2 macrophage polarization. **(D)** M2 (CD206+) macrophage cell ratio is depicted in the histogram. The mRNA levels of *Mrc1* in BMDM cells with different treatments. **(E)** Western blotting assay demonstrated phosphorylation of CSF1R and JAK2 inhibited by sulfatinib in RAW264.7 cells. **(F)** The levels of CSF1 protein in human OS cell culture supernatants after culture in different conditions for 24 h were assayed by enzyme-linked immunosorbent assay (ELISA). OS, osteosarcoma; BMDM, bone marrow-derived macrophage.

TAMs are typically present in M2 phenotypes, which are associated with tumor growth, angiogenesis, and metastasis. Previous studies have confirmed that the survival and differentiation of macrophages depend upon CSF1R signaling, which is a target of sulfatinib. To further verify its effect on macrophages or subsequent change of the TME, f irst, conditioned medium of mouse osteosarcoma cell line K7M2-wt was collected to evaluate the effects of cytokines secreted by tumor cells on macrophages. In contrast, stimulation of macrophages with either interleukin-13 (IL13) or IL4 induced M2-type macrophages. Therefore, macrophages derived from RAW264.7 cells were stimulated with IL13, IL4, and K7M2-CM with or without sulfatinib for 24 h; flow cytometry was used to measure the ratio of M1 and M2 macrophages by detecting the expression of surface markers CD80 and CD206 for M1 and M2 type macrophages, respectively. As expected, IL13, IL4, and K7M2-CM stimulation increased the proportion of M2 type, while sulfatinib significantly reduced the proportion of polarized M2 type macrophages. Moreover, the mRNA expression of the M2 macrophage-specific gene *Mrc1* was quantified by RT-qPCR and demonstrated similar results (**Figures 7A, B**). Moreover, primary BMDMs were isolated from mice to repeat the above experiment, which obtained the same results that BMDM polarization toward M2 type was accomplished by stimulation with IL13, IL4, and K7M2-CM and reduced by sulfatinib treatment (**Figures 7C, D**). These results suggest that OS-TME could promote M2 macrophage polarization while sulfatinib could suppress M2 polarization in OS-TME. To further investigate the role of CSF1R and its inhibitor in M2 polarization, the level of protein expression of RAW264.7 cells was detected after being stimulated with IL13 or IL4 and treated with sulfatinib simultaneously for 24 h. It was found that sulfatinib inhibited the phosphorylation of CSF1R and the phosphorylation of JAK2, which is consistent with previous studies that JAK2 is involved in M2 polarization (**Figure 7E**). To determine whether a similar microenvironment existed in human osteosarcoma, HOS and MG63 cells were cultured under serum-free and hypoxia conditions; the concentration of human CSF1 in the supernatant was detected by ELISA, which shows that CSF1 secretion increased under hypoxia and starvation conditions (**Figure 7F**). Therefore, it was speculated that sulfatinib could inhibit tumor growth by suppressing M2 macrophage polarization in the TME.

### 3.7 Sulfatinib inhibited tumor growth by suppressing M2 macrophage polarization

**Figure 8:**
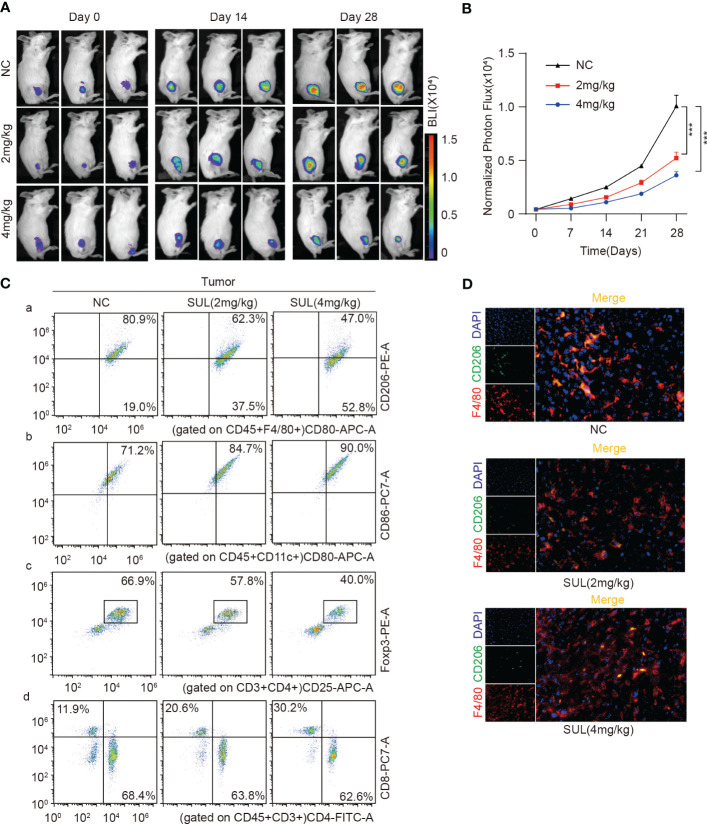
Sulfatinib inhibited tumor growth by suppressing M2 macrophage polarization. **(A)** Representative graph of luminescence intensity from the primary tumors of each treatment group measured on days 14 and 28 using an IVIS. **(B)** Monitoring of luminescence intensity from the primary tumors of each treatment group every 7 days. Data were expressed as normalized BLI (mean ± SD, ***p < 0.01). **(C)** Flow cytometry analysis of M2, DC, Tregs, and CD4/8+ T cells isolated from tumor tissues by *ex vivo* dissociation. **(D)** Immunofluorescence staining of TAM in *ex vivo* tumor tissues (scale bar, 100 μm). IVIS, *in vivo* fluorescence imaging system; BLI, bioluminescence imaging; TAM, tumor-associated macrophage.

To better monitor tumor growth, K7W2 cell lines were constructed with stable luciferase expression, and K7M2-Luc cells were inoculated into the tibial marrow cavity of BALB/c mice. Immunocompetent mice were used for further evaluation of the immune microenvironment *in vivo*. Once the tumors were measurable by *in In-vivo* fluorescence imaging system (IVIS), the mice were randomly assigned to three groups (n = 8 for each group) and received daily oral treatment with 2 or 4 mg/kg of sulfatinib or vehicle for 4 weeks. Mice were monitored and imaged every week thereafter, and bioluminescence imaging (BLI) was performed to detect tumor growth in the legs and distant metastasis. Compared with the control group, the BLI of orthotopic transplantation tumors was significantly decreased by sulfatinib treatment and more significant with the increasing dose (**Figures 8A, B**). However, no fluorescence was detected in the lung area in all groups until the end of the experiment, and no macroscopic metastatic nodules were found in the lung tissue, making it impossible to evaluate distant metastasis. To provide a better understanding of the effect of sulfatinib on the TME, the proportion of main immune cell compositions was preliminarily investigated by fluorescence-activated cell sorting (fluorescence-activated cell sorting (FACS) analysis. Single-cell suspensions of dissected tumors, the lungs, and the spleens were prepared by grinding the tissues through cell strainers, labeled with a combination of fluorescent antibodies, and analyzed by flow cytometry to determine different immune populations. As demonstrated in the flow cytometry plots, sulfatinib decreased the tumor- infiltrated M2 macrophages and was more significant with increasing dose, which was further confirmed by *ex vivo* immunofluorescence staining (**Figures 8C, D**). The proportion of tumor- infiltrated dendritic cells (DCs) and effector CD8+ T cells increased, while the proportion of immunosuppression- related Tregs decreased in the sulfatinib treatment group, and the proportion changes became more obvious as the dose increased. In accordance with the primary tumors, the proportion of M2 macrophages and myeloid-derived suppressor cells (MDSCs) in lung- infiltrated immune cells equally decreased, and the proportion of CD8+ T cells in the spleen increased following sulfatinib treatment in a dose-dependent manner (**Figures S3A, B**). Hence, we can infer that sulfatinib depolarized TAMs, increased antigen presentation capacity of DCs, diminished Tregs and MDSCs to reverse the immunosuppression of the TME, and improved the infiltration of CD8+ effector T cells, resulting in an effective anticancer immune response.

## 4 Discussion

Presently, there remains a percentage of refractory osteosarcoma; although many clinical trials of new drugs have been conducted, the overall outcome of osteosarcoma patients has not successfully improved (40, 41). Therefore, new treatments and drugs are urgently needed, especially for chemo-resistant osteosarcoma. Notably, effective targeted therapies are based upon specific mutations or amplifications in the primary tumor that are directly related to the tumorigenesis, such as human epidermal growth factor receptor 2 (HER2) amplification in breast cancer and epithelial growth factor receptor (EGFR) mutation in lung cancer (42, 43). In addition, multi-targeted TKIs not only inhibit tumor proliferation but also play a dual role in inhibiting angiogenesis and the pro-tumor microenvironment. Unfortunately, the clinical results of these drugs such as sorafenib and regorafenib in osteosarcoma treatment are not ideal, with a median progression-free survival time of 4–6 months (7, 8). We described preclinical studies of a novel multi-targeted tyrosine kinase inhibitor that inhibits multiple protein kinases, targeting tumors and the microenvironment. Sulfatinib can play an anti-tumor role by inhibiting tumor cell proliferation, enhancing chemosensitivity, inducing cell apoptosis, inhibiting the migration and invasion induced by cytokines, blocking differentiation of CAFs and polarization of TAMs, and modulating tumor immune microenvironment for more effective anti-tumor results.

Currently, studies have confirmed that targeted drugs combined with chemotherapy drugs significantly improved anti-tumor efficacy, which even has been adopted as a first-line regimen. FGFR1 is a member of the FGF receptor family, which is generally associated with tumor invasion, chemotherapy resistance, and poor prognosis (44). Targeting FGF/FGFR signaling pathway, either alone or in combination with chemotherapy, might be an effective strategy for treating liver and pancreatic cancers (45, 46). Previous research has shown that cytokines play a pleiotropic role in the tumor microenvironment as well as cancer drug resistance. bFGF promotes chondrosarcoma resistance to doxorubicin by upregulating the expression of X-ray repair cross-complementing protein 5 (XRCC5), while bFGF is the ligand of FGFR1 (47). Furthermore, FGFR1 is highly expressed in osteosarcoma and is associated with poor prognosis. In this study, we found that sulfatinib inhibits the phosphorylation of FGFR1, and downstream kinases in osteosarcoma cells play key roles in the inhibition of proliferation, promoting apoptosis and enhancing chemo-sensitivity. In addition, both angiogenesis and immunosuppression in the tumor microenvironment play roles in chemotherapy resistance. As sulfatinib is a multi-targeted TKI, not only directly targeting cancer cells but also regulating the tumor microenvironment, it may enhance chemotherapy sensitivity through multiple mechanisms.

Moreover, the tumor microenvironment functions as a double-edged sword in tumor progression: it is not only a barrier but also a favorable “soil” promoting invasiveness. Cytokines in the TME bind to membrane surface receptors or target cells and trigger a cascade amplification effect, ultimately promoting the migration and invasion of tumor cells, a mong which tumor autocrine is one of the sources of cytokines, which can promote migration and invasion through self-amplification. For example, melanoma can secrete bFGF in an autocrine manner to facilitate tumor metastasis and drug resistance (48). Likewise, bFGF autocrine loop also plays a role in the phenotype and differentiation of osteosarcoma (49). In our study, sulfatinib blocked FGF/FGFR1 signaling pathway in a manner blocking self-amplification. Current studies have shown that FGF, hepatocyte growth factor (HGF), and insulin-like growth factor (IGF) promote cell migration by inducing EMT (50, 51). EMT is mainly characterized by reduced epithelial connections and increased interstitial markers, which is an important process of tumor metastasis (52). Sulfatinib suppresses migration and invasion by altering EMT phenotype by inhibiting phosphorylation of FGFR1 and reducing bFGF-induced phosphorylation of AKT, SRC, and ERK. These pathways also play a role in VEGF-induced angiogenesis, and its receptor VEGFR2 is also one of the targets of sulfatinib; hence, the anti-tumor effect could be enhanced by inhibiting angiogenesis (52). To sum up, bFGF is an important cytokine in osteosarcoma TME, and sulfatinib blocking endocrine effects of OS cells plays a dual role in inhibiting tumors.

Growing evidence supports the vital role of CAFs in tumor progression, which can promote tumor cell proliferation and modulate the TME by remodeling the extracellular matrix and secreting cytokines or other growth factors (53). The source of CAFs is diverse, and it has been demonstrated that a proportion of CAFs are derived from the differentiation of mesenchymal stem cells (MSCs) via the action of bFGF and TGFβ (54). As a result, bFGF promotes tumor progression not only by directly acting on tumor cells but also by modulating CAFs (55). The bone marrow is the primary location of MSCs, so there is no surprise that MSCs differentiated into CAFs in OS-TME. However, the question is whether there are any other stem cells in the solid bony matrix. In our study, we isolated SSCs in OS-TME, confirmed that they are similar to SSCs in normal bone, and confirmed their differentiation into CAFs in OS-CM. This means that there also exists modulation in the TME. Despite the controversy over CAFs, it is believed MSCs are pluripotent and that CAFs are also multipotent cells. Sulfatinib indeed inhibited bFGF- induced CAF phenotype and CAF activation in the transcriptional and protein levels, which suggested that targeting CAF is the indirect mechanism in which sulfatinib exerts its effective anti-tumor effects.

Inhibition of macrophage polarization by targeting CSF1R has been studied in other tumors in combination with radiotherapy, chemotherapy, and immunotherapy (56, 57). Pexidartinib (PLX3397), a CSF1R inhibitor, was approved by Food and Drug Administration (FDA) for the treatment of tenosynovial giant cell tumors (58). Moreover, the role of CSF1R in osteosarcoma has also been studied (59), but CSF1R is mainly expressed in TAMs rather than OS tumor cells. The prevalence of TAMs and their correlation with poor prognosis in osteosarcoma suggests that inhibitors of CSF1R are a reasonable agent in osteosarcoma treatment. In addition to the targeted inhibition of TAM, systemic application of sulfatinib can reduce FOXP3+ Tregs and increase DC cells and CD8+ T cells. M2 macrophages can convert CD4+ CD25 − T cells to Tregs and Tregs promote macrophage polarization in reverse, so the reduction of Tregs can be explained by inhibition of M2 macrophages. Likewise, by diminishing M2 macrophages, the adhesion of CD8+ T cells was reduced and the infiltration of CD8+ T cells was promoted by elevating CCL2, CXCL10, and other factors (60). Although no spontaneous lung metastasis was observed in this study, a similar proportion of immune cells were also confirmed in lung tissue. Moreover, FGFR inhibitors induce the reduction or disappearance of MDSCs from the TME partly by targeting cytokine- secreting CAFs. Therefore, as CSF1 and FGF signals are both involved in the accumulation of tumor- promoting M2 macrophages, MDSCs, and Tregs (44), the dual inhibition of CSF1R and FGFR1 by sulfatinib may be more effective for cancer therapy than selective CSF1R inhibition.

The limitation of this study is the “translational gap” between animal experiments and clinical application. Moreover, the heterogeneity of individual samples should be considered. In order to partially make up some of these deficiencies, we combined *in vivo* models and bioinformatics analysis. In addition, parallel methods were used for the validation of each assay.

**Figure 9:**
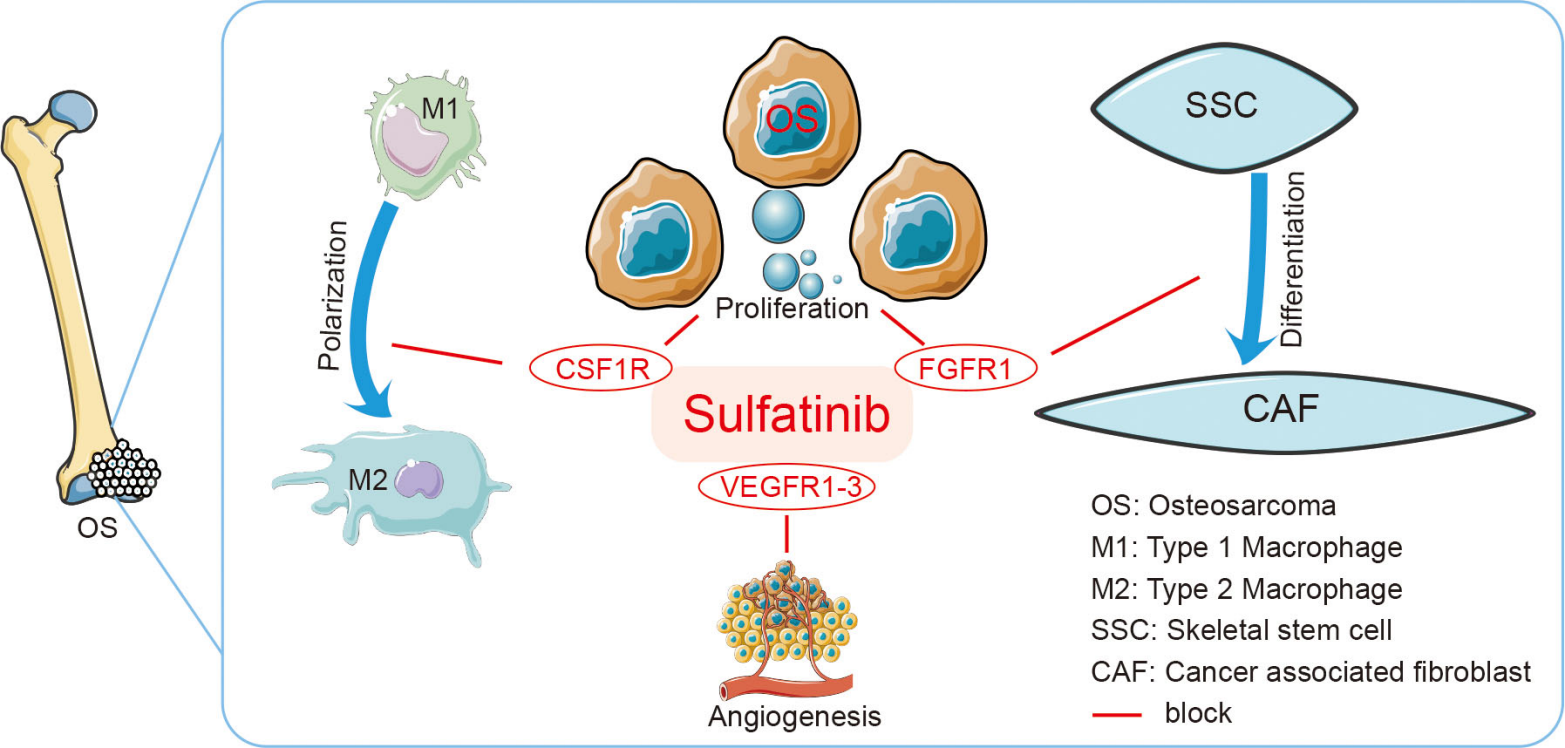
Graphic Abstract Sulfatinib is a multi-targeted tyrosine kinase inhibitor of FGFR1, CSF1R, and VEGFR1–3. It suppresses osteosarcoma by inhibiting proliferation, migration, and invasion of tumor cells and inhibiting differentiation of SSCs to CAFs and polarization of TAMs to modulate immune microenvironment. SSCs, skeletal stem cells; CAFs, cancer-associated fibroblasts; TAMs, tumor-associated macrophages.

Overall, we found that sulfatinib demonstrated anti-tumor effects of osteosarcoma in xenograft models as well as orthotopic implanted mice models. The drug can significantly inhibit tumor proliferation, migration, and invasion as well as angiogenesis and inhibit tumor growth by modulating the tumor immune microenvironment (**Figures 9**). However, the practical application of sulfatinib needs to be further verified, and it has been applied to extra pancreatic neuroendocrine tumor treatment. Toxicity and anti-tumor effect need to be further evaluated combined with either chemotherapy or immunotherapy.

## Data availability statement

Publicly available datasets were analyzed in this study. This data can be found here: https://www.cancer.gov/about-nci/organization/ccg/research/structural-genomics/tcga https://ocg.cancer.gov/programs/target.

## Ethics statement

The animal study was reviewed and approved by Animal Care and Use Committee of the Chinese PLA General Hospital.

## Author contributions

LS and BW: conceptualization. XM and BW: supervision. LS and LJ: investigation. LS and GS: methodology. BJ and HY: data analysis. LS and LJ: writing. GS, WY, and HX: resources. All authors contributed to the article and approved the submitted version.
