# Supplementary material for: Sulfatinib, a novel multi-targeted tyrosine kinase inhibitor of FGFR1, CSF1R, and VEGFR1–3, suppresses osteosarcoma proliferation and invasion via dual role in tumor cells and tumor microenvironment
Source: Front Oncol. 2023 Jun 8;13:1158857. doi: 10.3389/fonc.2023.1158857 (PMC10286821; doi:10.3389/fonc.2023.1158857)
Supplement: Supplementary file 1 [file DataSheet_1.pdf]

Supplementary Material

Sulfatinib, a novel multi-targeted tyrosine kinase inhibitor of FGFR1, CSF1R, VEGFR1-3, suppresses osteosarcoma proliferation and invasion via dual role in tumor cells and tumor microenvironment

Liao Song, Li Jianxiong, Gao Song, Han Yuchen, Han Xinli, Wu Yanan, Bi Jingyou, Xu Meng*, Bi Wenzhi*

Liao Song and Li Jianxiong contributed equally to this work.

***Correspondence:** Bi Wenzhi:Biwenzhi@sina.com;
Xu Meng:13501175839@163.com

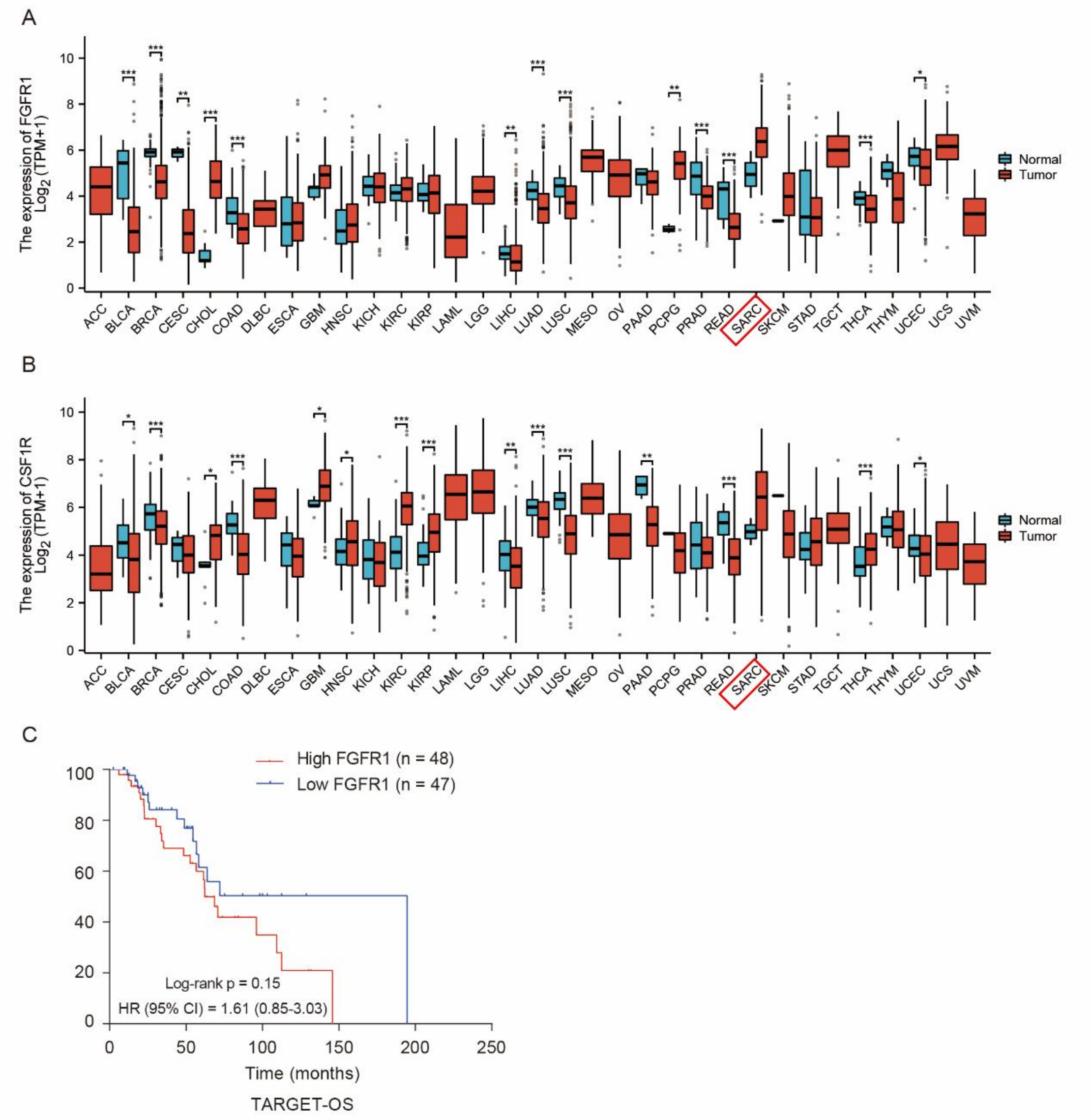

Supplemental Figure 1 FGFR1 and CSF1R expression are increased in sarcoma.

A. FGFR1 expression is increase in sarcoma from analysis of TCGA data.

B. CSF1R expression is increase in sarcoma from analysis of TCGA data.

C. Survival analysis of TARGET data indicates the high expression of FGFR1 correlate with poor prognosis in OS patients.

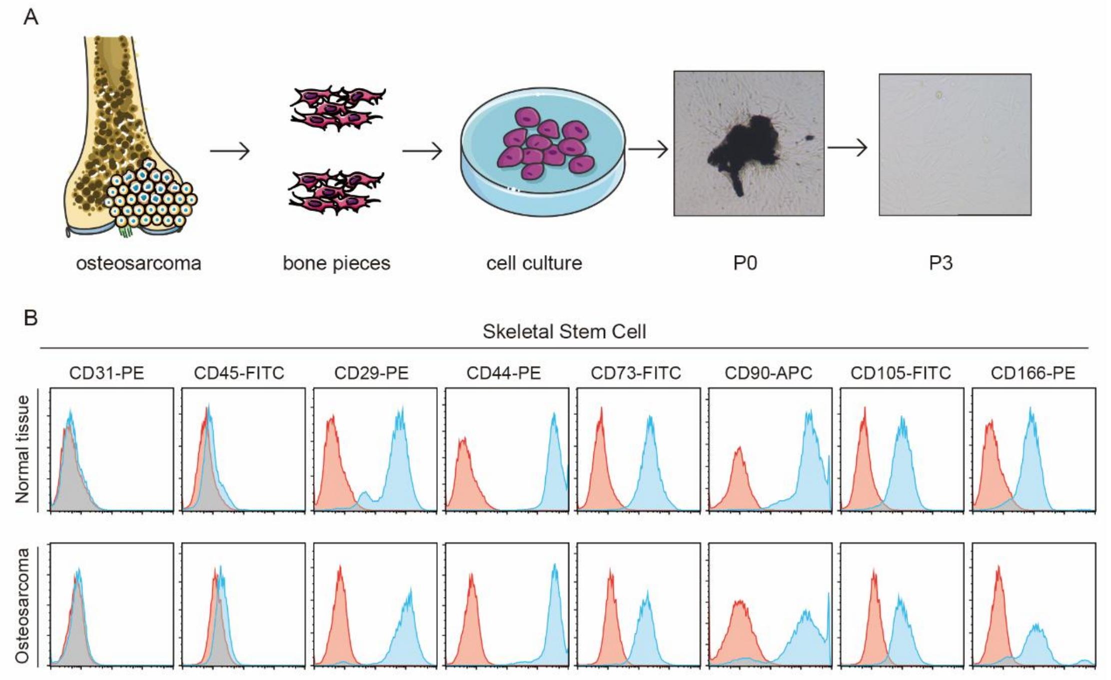

Supplemental Figure 2 Isolation and identification of skeletal stem cell.

A. A schematic workflow of skeletal stem cells (SSCs) isolation.

B. SSCs isolated from the tumor tissue and adjacent normal bone have the same stem cell markers.

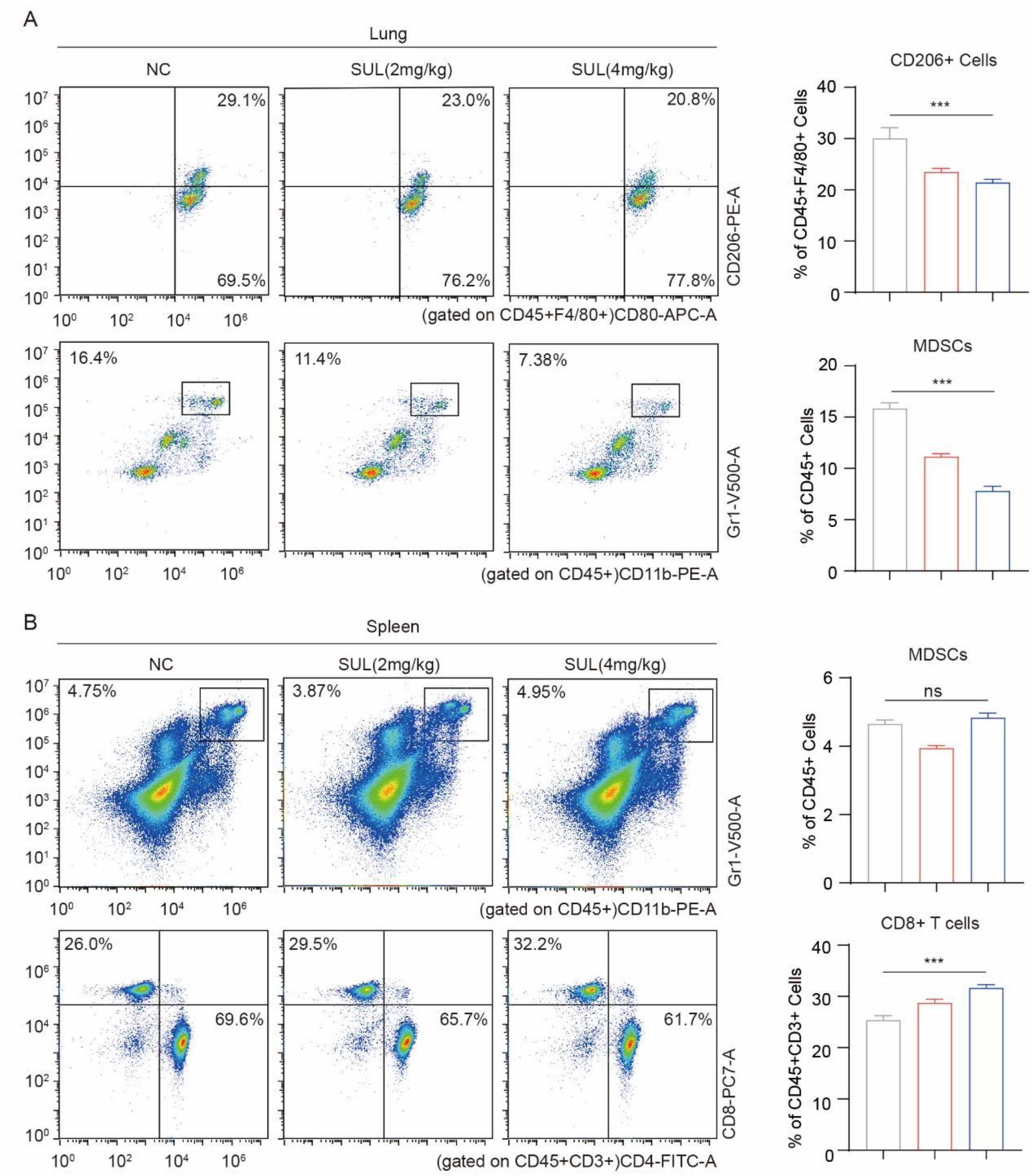

Supplemental Figure 3 Proportion of immune cells in lung and spleen after treatment with sulfatinib.

A. Flow cytometry analysis of M2 macrophages and MDSCs isolated from lung tissues by ex vivo dissociation.

B. Flow cytometry analysis of MDSCs and CD4/8 T cells isolated from spleen tissues by ex vivo dissociation.
